# Zinc Acetate as a Cross-Linking Agent in the Development of Enteric Microcapsules for Posaconazole

**DOI:** 10.3390/pharmaceutics17030291

**Published:** 2025-02-22

**Authors:** Marta Szekalska, Giedrė Kasparavičienė, Jurga Bernatonienė, Eliza Wolska, Paweł Misiak, Karolina Halina Markiewicz, Agnieszka Zofia Wilczewska, Anna Czajkowska-Kośnik, Katarzyna Winnicka

**Affiliations:** 1 Department of Pharmaceutical Technology, Medical University of Białystok, Mickiewicza 2C, 15-222 Białystok, Poland; anna.czajkowska-kosnik@umb.edu.pl (A.C.-K.); katarzyna.winnicka@umb.edu.pl (K.W.); 2 Department of Drug Technology and Social Pharmacy, Faculty of Pharmacy, Medical Academy, Lithuanian University of Health Sciences, Sukileliu pr. 13, LT-50161 Kaunas, Lithuania; giedre.kasparaviciene@lsmu.lt (G.K.); jurga.bernatoniene@lsmu.lt (J.B.); 3 Department of Pharmaceutical Technology, Medical University of Gdańsk, Hallera 107, 80-416 Gdańsk, Poland; eliw@gumed.edu.pl; 4 Department of Polymers and Organic Synthesis, Faculty of Chemistry, University of Białystok, Ciołkowskiego 1K, 15-245 Białystok, Poland; p.misiak@uwb.edu.pl (P.M.); k.markiewicz@uwb.edu.pl (K.H.M.); agawilczuwb@gmail.com (A.Z.W.)

**Keywords:** microcapsules, sodium alginate, pectin, posaconazole, cross-linking, co-extrusion

## Abstract

**Background/Objectives:** Posaconazole is an antifungal agent from triazoles with variable bioavailability. To avoid its irregular absorption caused by gastric conditions and ensure more repeatable pharmacokinetic enabling the maximization of its absorption regardless of food intake without the need to administer multiple doses, can be provided by the technology of enteric drug preparations. The cross-linking of polysaccharide polymers with divalent and trivalent cations enables multi-unit formulations to be obtained that prevent drug absorption in the stomach. Microcapsules, as an example of multi-unit drug dosage forms, provide more predictable gastric emptying, depending on nutritional status, and spread extensively throughout the gastrointestinal tract. **Methods:** Therefore, the utilization of zinc acetate for the cross-linking of the alginate and pectin mixture was evaluated. The obtained formulations were evaluated for the impact of cross-linking process and pectin’s presence on their pharmaceutical, mucoadhesive, physicochemical and antifungal properties. **Results:** It was shown that cross-linked microcapsules by zinc acetate provided delayed posaconazole release. Additionally, the cross-linking process with Zn^2+^ ions significantly enhanced antifungal activity against the analyzed *Candida* strains. It was observed that pectin content in the formulation enhanced the swelling ability in an intestinal condition and increased the mucoadhesive properties of drug-loaded formulations to the intestinal mucosa.

## 1. Introduction

Multi-compartment drug dosage forms are a relatively innovative strategy in pharmaceutical technology regarding the distribution of a drug substance in separate, multiparticulate carriers. In comparison to single-unit formulations, they ensure balanced drug release, prevent a sudden dose ejection, and reduce the risk of local irritation. Therefore, for drugs for which enteric release is desirable, multi-unit formulations are recommended [1,2]. An example of multi-compartment drug forms is microcapsules, which consist of a core containing the drug substance and a polymer shell [3].

Posaconazole (POS) is an antifungal agent from the second-generation group of triazoles. POS interferes with the cell membrane’s function by inhibiting 14 α-demethylase (CYP51), thereby preventing the synthesis of ergosterol. It is characterized by a broad spectrum of activity, including *Candida* and *Cryptococcus*, *Aspergillus*, *Fusarium* spp., *Zygomycetes*, and endemic fungi [4,5]. POS is characterized by variable bioavailability, which is significantly related to its low water solubility. In addition, POS pharmacokinetics also depends on the presence and quality of the food intake of patients and their inter-individual differences [6].

Sodium alginate (ALG) is a natural polysaccharide polymer that exhibits a lack of toxicity, biodegradability, and biocompatibility. Polymers possess many advantages, including gelling, swelling, and adhesive properties. ALG’s ability to produce gel formation in contact with cationic ions is often utilized in enteric drug formulation technology [7]. ALG modified in the cross-linking process enables formulations that are insensitive to gastric acid to be obtained, which pass into the intestinal environment unchanged. In the intestine environment, the higher concentration of phosphate ions causes the ALG matrix to relax and ensures intestinal drug release [7,8,9].

Despite the many advantages of ALG, the application of this polymer in drug formulations is limited. It is related to the low flexibility, brittleness, and poor mechanical properties of ALG. Hence, new technologies to improve the characteristics of ALG formulations are needed. There are reports regarding the advantageous combination of ALG and pectin (PEC). PEC is found naturally in the cell walls of different plant species. It belongs to the group of natural polysaccharides with mucoadhesive, swelling, and gelling properties. Heteropolymers based on a combination of ALG and PEC might possess the advantages of each distinct polymer. These include a rise in the percentage of active drug encapsulation due to the synergistic interaction with Ca^2+^ ions. In addition, it can enable the creation of more elastic gels with tolerance to a wide range of conditions, including pH and temperature [10,11].

Co-extrusion is one of the widely applied physicomechanical methods for microcapsule preparation. It involves pumping a stream of liquid and core material through small tubes or needles. Next, the drops are introduced into a solution containing divalent cations, and the shell polymer undergoes a cross-linking process. The use of a syringe pump for extrusion facilitates the work and provides stable parameters during the experiment [12].

The aim of the presented study was to apply zinc ions to the cross-linking of ALG and PEC combinations for the first time to develop enteric multi-unit drug dosage forms—microcapsules with POS using the co-extrusion technique. Zn^2+^ ions were used as the cross-linking factor due to their extensive interaction with polymers. Moreover, they exhibited an ability to impede the expansion of bacteria, yeast, and fungi [13,14]. In the next stage, the quality of received microcapsules and the effect of the concentration of Zn^2+^ ions and PEC addition on the pharmaceutical properties were assessed. Additionally, the antifungal activity of all formulations against *Candida strains C. albicans*, *C. krusei*, and *C. parapsilosis* was evaluated.

## 2. Materials and Methods

Sodium alginate (ALG) used in our study was composed of 39% guluronic acid (G) and 61% mannuronic acid (M) (M/G ratio of 1.56) and possessed a molecular mass of 3.5 × 10^5^ Da, acquired from *Macrocystis pyrifera* and procured from Merck (Darmstadt, Germany). The viscosity of 1% ALG solution at 25 °C was indicated as 282 mPa∙s. Zinc acetate dihydrate (Zn(OAc)_2_·2H_2_O), acetonitrile, methanol, and polysorbate 80 were also obtained from Merck (Darmstadt, Germany). Low methoxylated pectin (PEC) (26% esterification, 24% degree of amination, 90% galacturonic acid content, 3.8 pH value of 2.5% solution prepared using distilled water at 20 °C) was obtained by Herbstreith & Fox and GmbH & Co. KG (Neuenbürg, Germany). Posaconazole (POS) was acquired from Kerui Biotechnology Co., Ltd. (Xi’an, China). Pure sodium phosphate tribasic dodecahydrate and hydrochloric acid were obtained from Chempur (Piekary Śląskie, Poland). Water was procured by the Milli-Q Reagent Water System (Billerica, MA, USA). Cultures of *Candida albicans* ATCC^®^ 10231, *Candida krusei* ATCC^®^ 6528, and *Candida parapsilosis* ATCC^®^ 22019 were obtained from the American Type Culture Collection, and Sabouraud dextrose agar was purchased from Biomaxima (Lublin, Poland). All applied filters (0.45 µm) were procured from Millipore (Billerica, MA, USA) and Alchem (Toruń, Poland). Filtered paper was procured in Chemland (Stargard, Poland). Porcine stomachs and small intestine mucosa were received from a local abattoir (Turośń Kościelna, Poland). The agreement of the Local Ethical Committee for Experiments on Animals for this procedure was not necessary. The mucosal fragments were frozen at −20 °C for up to 30 days. All other components were of laboratory grade.

### 2.1. Development of ALG and ALG/PEC Microcapsules

In the first stage, an appropriate amount of ALG was added to water and stirred via a mechanical stirrer (DT 200, Witko, Łódź, Poland) to acquire a uniform solution. Thereafter, part of the ALG solution was connected with the PEC solution. To receive a drug-loaded formulation, POS was solubilized in polysorbate 80 and then was gradually suspended in the ALG and ALG/PEC dilutions. The composition of the placebo and POS-loaded formulation is described in Table 1. Microcapsules were created using the extrusion technique. The medical syringe containing the mixture was placed into the microcapsule pump (Landgraf Laborsysteme, Langenhagen, Germany). The emulsion was dripped through the needle into the zinc acetate solution and applied as a cross-linking agent, which was continuously stirred using a magnetic stirrer IKA Combimag RET (IKA, Warsaw, Poland) at 200 rpm. The microencapsulation pump dripping rate was 0.7 mL/min. In order to properly form a polymer wall, the solution with the microcapsules was mixed for 20 min. All formulations were isolated on filter paper, rinsed with distilled water, and then dried at room temperature (22 ± 2 °C) by day.

### 2.2. Attributies of ALG and ALG/PEC Dilutions

The viscosity of the placebo of ALG and ALG/PEC solutions and solutions containing POS was assessed 24 h after preparation using a Brookfield DV-III ULTRA Viscometer (Stuttgart, Germany). The viscosities of the solution samples (0.5 g) were evaluated with a shear rate of 2 s^−1^. The CP-52 spindle was applied. The experiment was carried out at 25 ± 1 °C. pH determination of the ALG and ALG/PEC dilutions and ALG and ALG/PEC mixtures with POS was assessed at 25 ± 1 °C by applying the pH metre Orion 3 Star (Thermo Scientific, Waltham, MA, USA).

### 2.3. Microcapsules Quality Assessment

#### 2.3.1. Scanning Electron Microscopy (SEM)

Microcapsules after gold sputtering (2 nm layer) were imaged using a scanning electron microscope (SEM) Phenom Pro G5 (Phenom World, Eindhoven, The Netherlands) was fitted with a backscattered (BSD) detector (detection at 5–10 kV and with distance involving 8 mm).

#### 2.3.2. Microcapsules Weight and Diameter Uniformity

The average weight and diameter of received and dried microcapsules were calculated to evaluate the microcapsules’ uniformity. The diameter of ten randomly selected microcapsules in each formulation was measured using the Digital Calliper micrometre (BGS Technic, Wermelskirchen, Germany). The percentage of the moisture level was analyzed by balance to assess moisture (Radwag WSP 50SX, Radom, Poland).

#### 2.3.3. Homogeneity of Active Substance Ingredient, Encapsulation Efficiency, and Production Yield

In total, 10 mg of microcapsules in a volumetric flask containing 100 mL 0.1 M HCl pH 1.2 were inserted into a water chamber at 37 ± 0.5 °C with 50 rpm for a day. The obtained mixtures were stirred, filtered, and analyzed with a UV-Vis spectrophotometer (Genesys 10S, Thermo Scientific, Madison, WI, USA) at a wavelength of 260 nm.

The active substance in microcapsules (L) was determined by the following expression:

L = Q_m_/W_m_ × 100 (1)

where Q_m_ is the active substance in the formulation, and W_m_ is the formulation weight.

The active substance encapsulation efficiency (EE) was appointed by the following formula:

EE = Q_a_/Q_t_ × 100 (2)

where Q_a_ is the POS content, and Q_t_ is the assumed POS content.

The yield of production (Y) was computed by the following equation:

Y = W_m_/W_t_ × 100 (3)

where W_m_ is the microcapsule mass, and W_t_ is the assumed mass of the drug and polymer [15].

#### 2.3.4. Swelling Ability

The swelling ability of the developed microcapsules was expressed as a swelling ratio (SR) and was analyzed in media characterized by different pH levels: pH 1.2 (0.1 M HCl) and pH 6.8 (phosphate buffer, PBS). In total, 20 mg of microcapsules was placed in the baskets from the USP dissolution device. Then, they were added to containers with a 15 mL medium. Tests were carried out at the temperature of 37 ± 1 °C and at intervals (5, 10, 15, 30, 60, 90, 120, 180 and 240 min). After the passage of time, microcapsules were cautiously drained and weighted. In the next step, the swelling ratio was determined by the following formula:

SR = (W_s_ − W_0_)/W_0_ (4)

where W_0_—pre-weight of microcapsules; W_s_—swelling weight of microcapsules [16].

#### 2.3.5. Firmness and Mucoadhesiveness

Both firmness force and mucoadhesiveness were assessed by applying Texture Analyzer TA.XT.Plus (Stable Micro Systems, Godalming, UK). The firmness force of the sample containing ten freshly prepared microcapsules was determined with the P/100 plate applied as a probe with a maximum device force of 6500 g. The mucoadhesive ability (the work of mucoadhesion (W_ad_) and the maximum detachment force (F_max_)) of the microcapsules were evaluated using porcine stomach and intestine mucosa as adhesive layers. The microcapsules were subjected to hydration in 500 µL of 0.1 M HCl (pH 1.2) or in phosphate buffer (PBS, pH 6.8) for 15 min before testing [17]. The process conditions included velocity at the following levels: pretest—0.5 mm/s; test and post-test—0.1 m/s. The contact time of microcapsules with mucosa was 120 s with an applied strength of 1 N.

#### 2.3.6. Posaconazole In Vitro Release Evaluation

The in vitro POS release assay was determined by USP apparatus type II (Erweka Dissolution Tester Type DT 600HH, Heusenstamm, Germany). Method A using a pH-change technique, according to the monograph “Delayed-release solid dosage forms”, was applied [18]. The acid stage was carried out by placing microcapsules containing 100 mg of POS into 750 mL of 0.1 M HCl. After 2 h, the medium was completed with 250 mL of 0.2 M phosphate buffer (PBS), and the buffer stage was conducted. A total of 1% sodium dodecyl sulphate (SDS) was added to the PBS to acquire *sink* criteria. As a control, the pure drug in the dose corresponding to its presence in the microcapsules was applied. The POS content in the release solution was determined by the spectrophotometer Genesys 10S UV-Vis (Thermo Scientific, Madison, WI, USA) at the wavelength of 260 nm and calculated according to the reference calibration curve (with linearity in the concentration 1–20 μg/mL and a correlation value (R^2^) of 0.999). To analyze the mechanism of POS release, the data were assessed with mathematical equations [19].

#### 2.3.7. Antifungal Activity Evaluation

The antifungal action of the microcapsules was carried out by the plate diffusion method [20]. For the analysis of *Candida* cells, *Candida albicans* ATCC^®^ 10231, *Candida krusei* ATCC^®^ 6528, and *Candida parapsilosis* ATCC^®^ 22019 were applied in a concentration of 0.5 in McFarland (5 × 10^6^ CFU/mL) in sterile 0.9% NaCl and were labelled by a turbidity detector (Densitometer DEN-1B, Biosan, Riga, Latvia). *Candida* inoculum (50 µL) was applied to the Sabouraud dextrose agar in Petri dishes. Then, 20 mg of microcapsules was placed in an agar well (5 mm diameter). As the control, 50 μL of POS/DMSO dilution was added to an agar well. Dishes were cultured for 24 and 48 h at a temperature of 37 ± 0.1 °C. The suppression areas (mm) were assessed by an electronic calliper (Mitutoyo, Kawasaki, Japan).

#### 2.3.8. Perform a Thermal Analysis

Thermal analysis was performed using two methods: thermogravimetric (TGA) and differential scanning calorimetry analysis (DSC). The analysis of the pure formulation compounds was performed as follows: ALG, PEC, POS, Zn(OAc)_2_·2H_2_O, empty microcapsules (P1-P6), and microcapsule formulations with POS (F1-F6) were carried out using a Mettler Toledo Star TGA/DSC unit. Weighted samples, 3–5 mg, were applied in aluminum oxide crucibles and heated (from 25 °C to 480 °C in TGA analysis and from 25 °C to 200 °C in DSC assay) under an argon flow. Probes were heated at 10 °C/min. After this process, samples were cooled to 25 °C with a velocity of −20 °C/min. A blank pan was utilized as a reference.

#### 2.3.9. Attenuated Total Reflectance–Fourier Transform Infrared Spectroscopy (ATR–FTIR)

The ATR–FTIR spectra of the pure ALG, PEC, POS, Zn(OAc)_2_·2H_2_O, empty microcapsules (P1–P6), and microcapsule formulations with POS (F1–F6) were examined by a Thermo Scientific Nicolet 6700 FTIR spectrophotometer (Waltham, MA, USA). The data were recorded within 4000 to 500 cm^−1^ of the wavenumber range by averaging 32 scans with a resolution of 4 cm^−1^ and normalized against the background spectrum.

### 2.4. Statistical Analysis

The acquired data are featured as mean values with standard deviations (mean ± SD). Statistical analysis was conducted with StatSoft Statistica 13.3 software (StatSoft, Kraków, Poland). Before conducting statistical analysis, the normality of the data was verified with the Shapiro–Wilk test. The results from the firmness, size, mucoadhesive, and fungi activity studies did not follow a normal distribution, and they were assessed by the Kruskal–Wallis test. Measurements were regarded as significant at *p* < 0.05.

## 3. Results

**Figure 1:**
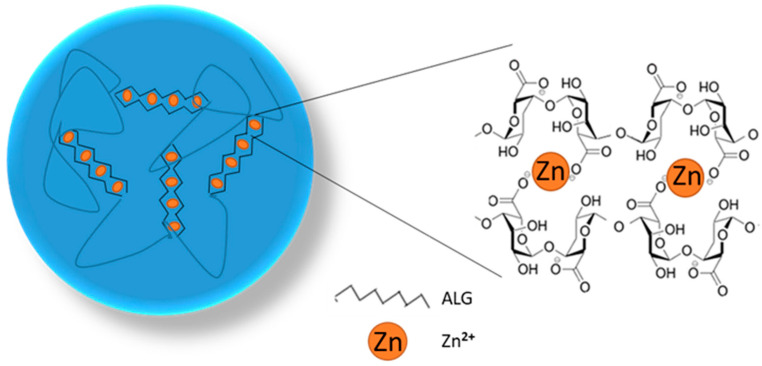
Scheme of ALG cross-linking by applying Zn^2+^ ions in the “egg box” process.

ALG and PEC are polymers from the group of natural polysaccharides, which are characterized by their capacity to form three-dimensional and stable network gels in the gel formation process according to the “egg box” model. This mechanism is related to the carboxyl group interactions of ALG guluronic acid monomers (Figure 1) and the galacturonic acid present in PEC with divalent metal ions such as Ca^2+^, Zn^2+^, and Ba^2+^ or trivalent metals such as Fe^3+^ and Al^3+^ [21]. The selection of the cross-linking factor type and its concentration has a major influence on the formulation properties. There are reports of the greater interaction of Zn^2+^ cations with ALG, resulting in more extensive cross-linking and prolonged drug release profiles than in the case of Ca^2+^ ions [21,22]. Additionally, Zn^2+^ ions enabled the creation of a stronger PEC network with greater stability in the upper gastrointestinal tract [23]. Moreover, Zn^2+^ ions possess documented antifungal properties, which might have a positive effect on the activity of POS-loaded microcapsules [13,14,22,24].

As the diameter of the needle used in the co-extrusion technique makes it impossible to obtain microcapsules using high-viscosity solutions, a 2% solution of ALG characterized by optimal properties was selected for the study. The obtained ALG and ALG/PEC solutions and ALG and ALG/PEC solutions containing POS were subjected to viscosity and pH evaluation. It was found that PEC and POS addition resulted in a reduction in the pH value and viscosity of the ALG solution (Table 2).

### 3.1. Quality Estimation of ALG and ALG/PEC Microcapsules

**Figure 2:**
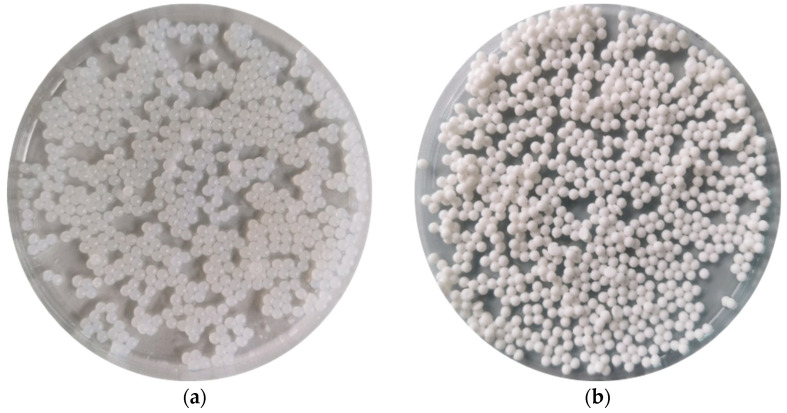
Representative images of microcapsules after preparation: (**a**) formulation P1 without an active substance and (**b**) formulation F1 with POS obtained with a camera.

**Figure 3:**
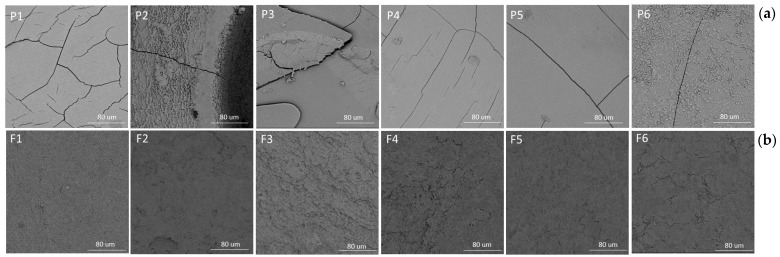
Representative SEM images of microcapsules: (**a**) formation of microcapsules without active substance (P1–P6) and formulation with POS (F1–F6) under magnification 120× and (**b**) surface microcapsules without drugs (P1–P6) and formulation with POS (F1–F6) under magnification 1000×.

It was deduced that the co-extrusion technique enabled spherical ALGs and ALG/PEC microcapsules cross-linked with zinc acetate to be obtained (Figure 2). SEM images indicated that the POS-loading of ALG and ALG/PEC microcapsules was characterized by a more regular shape than the placebo formulations (Figure 3a). Surface topography after sputtering with gold recorded at magnifications of 1000× showed that all microcapsules were rough with visible minor cracks (Figure 3b). Deepathomas et al., who prepared ALG beads cross-linked with zinc chloride, suggest that the ruptures might be related to the action of the electron beam used during the analysis [13]. Moreover, Nayak et al. implemented cracks that might be the result of the fractional breakdown of the network of polymers during the drying process [25].

**Figure 4:**
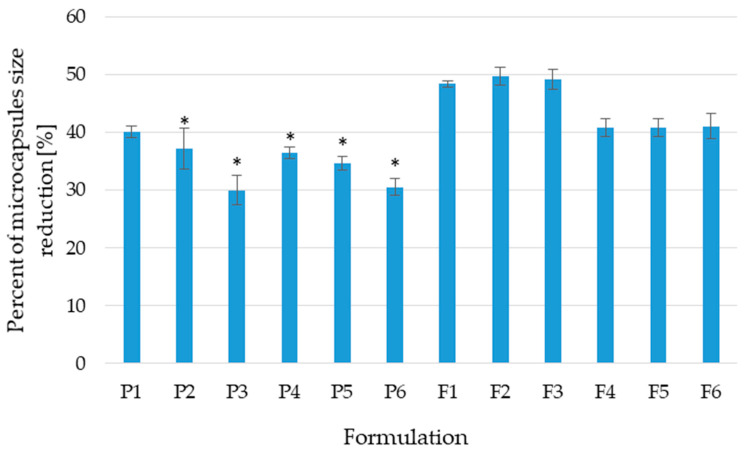
Variations in size between wet and dry microcapsules expressed as percentage of reduction in microcapsules’ diameter (mean ± SD, *n* = 10, * significant statistical difference at (*p* < 0.05) compared to the correspondent value of F1).

The diameter of wet and dry particles is one of the key parameters that provide information about the impact of the composition and production process on the product’s final size. In the case of the placebo formulation, it was observed that the utilization of a cross-linking factor contributed to a reduction in the difference in the particle’s diameter. Moreover, for all the formulations, the presence of PEC protected the microcapsules from shrinking (Figure 4). Quality analysis expressed that the diameter of wet and dry microcapsules for the placebo and formulations with POS increased with the increase in the concentration of the cross-linking agent (Table 3). However, production yield, drug loading, and encapsulation efficiency decreased with the increase in the concentration of Zn^2+^ ions applied for cross-linking. In microcapsules with the presence of PEC, a reduction in size alongside increased drug loading and encapsulation efficiency was observed. The diameter tests demonstrated the uniformity of the microcapsules’ size within the formulation, which proved the high uniformity of the production process. The presence of moisture in the drug formulation is a relevant parameter affecting its stability and physical properties [26]. The received data expressed that microcapsules of all formulations were characterized by the absence of moisture (0%).

**Figure 5:**
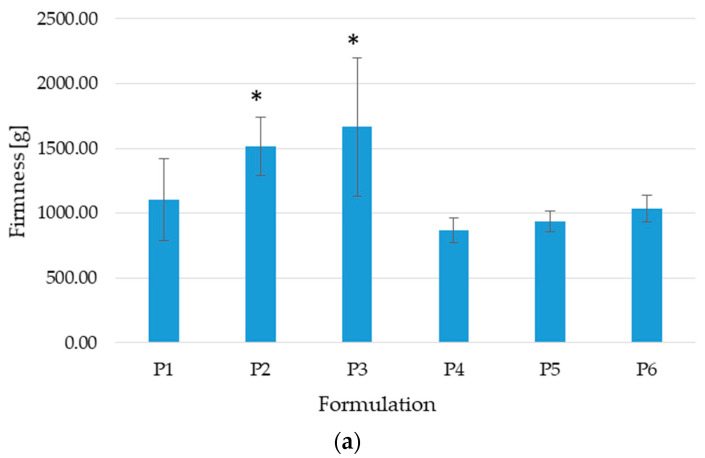

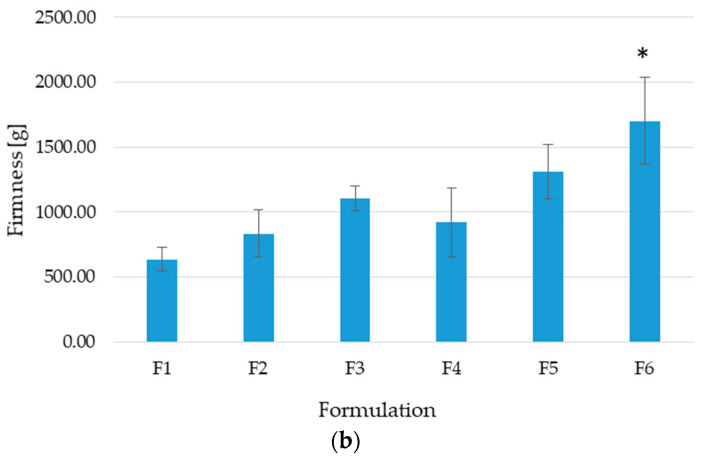
Firmness of microcapsules: (**a**) placebo (P1–P6) and (**b**) POS-loaded formulation (F1–F6) (mean ± SD, *n* = 3, * significant statistical difference at (*p* < 0.05) compared to correspondent value of F1).

The firmness of microcapsules is a factor indicating the breaking strength and is stated as the force needed to burst the microcapsules. The obtained data, in the placebo formulation and in POS-loaded microcapsules, expressed those formulations created using 5% zinc acetate required a higher force to break them (Figure 5). This was observed by Matulyte et al., who prepared calcium ALG microcapsules containing nutmeg essential oil. This proves that microcapsules cross-linked by 5% calcium chloride are more stable than formulations cross-linked by 2% calcium chloride [27]. Placebo formulations composed of ALG/PEC combinations showed lower crushing resistance (from 867.40 ± 96.30 g in formulation P4 to 1032.81 ± 103.27 g in P6). Interestingly, ALG/PEC microcapsules containing POS proved more resistant to disintegration than the POS-loaded ALG formulation—F1 vs. F4 (634.84 ± 92.33 vs. 919.37 ± 265.41) F2 vs. F5 (834.43 ± 180.97 g vs. 1309.48 *±* 208.54), and F3 vs. F6 (1103.58 ± 86.95 g vs. 1703.29 ± 334.13 g).

### 3.2. Swelling and Mucoadhesiveness

Swelling capacity is a relevant aspect of hydrophilic polymers applied in controlled drug delivery dosage forms. This ability significantly affects drug diffusion and drug release [28]. Additionally, swelling is an attribute enabling the initiation of the mucoadhesive process [29].

**Figure 6:**
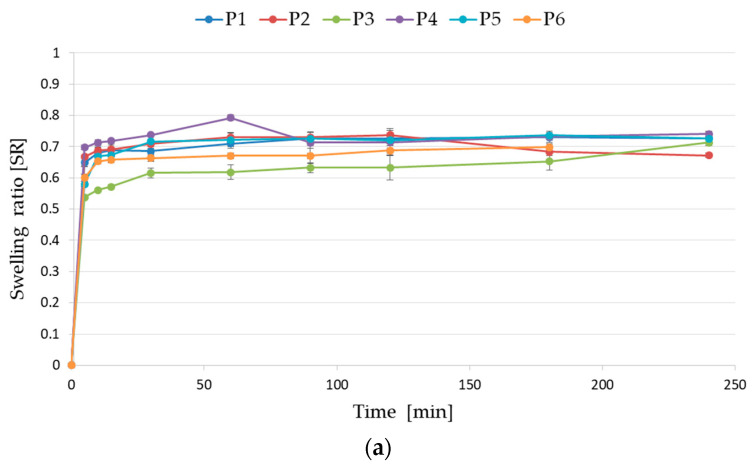

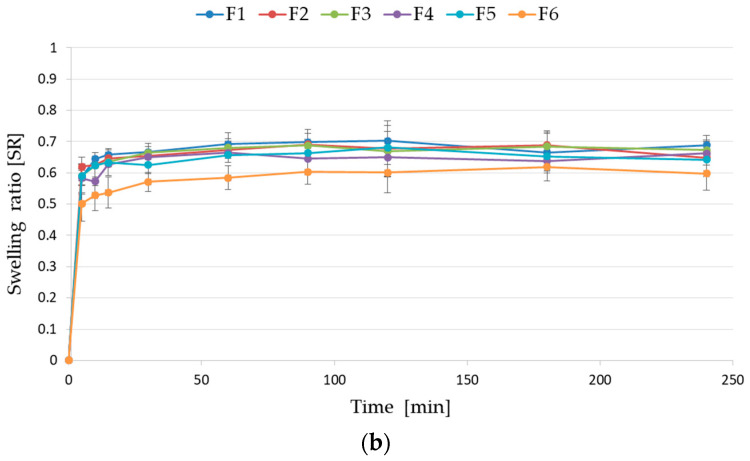
Swelling ratio of (**a**) empty formulation (P1–P6) and (**b**) formulation containing POS (F1–F6) microcapsules in HCl at pH 1.2 (mean ± SD, *n* = 3).

In order to mimic the stomach or intestinal terms, the swelling ratio of ALG and ALG/PEC microcapsules cross-linked by Zn^2+^ ions was explored in different media—0.1 M HCl (pH 1.2) and phosphate buffer (PBS, pH 6.8) (Figure 6). In acidic conditions, the highest swelling capacity (0.792 ± 0.009 after 60 min) was possessed by formulation P4—composed of ALG/PEC and cross-linked with 2% zinc acetate (Figure 6a). The lowest ability to absorb the acidic medium was achieved by formulation P3, which was composed only of ALG and was cross-linked with 5% zinc acetate. This formulation demonstrated the value 0.537 ± 0.001 after 5 min, then maintained a plateau throughout the study to reach a maximum of 0.713 ± 0.011 at 240 min (Figure 6a). In the case of formulations containing POS, all microcapsules proved to have similar swelling abilities, except the formulation F6, which was characterized by the lowest medium intake capacity reaching a maximum of 0.618 ± 0.044 after 180 min of the test (Figure 6b).

**Figure 7:**
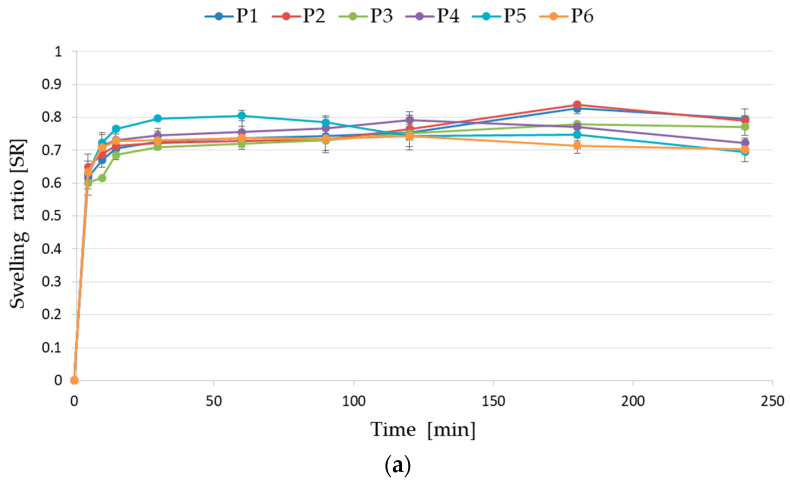

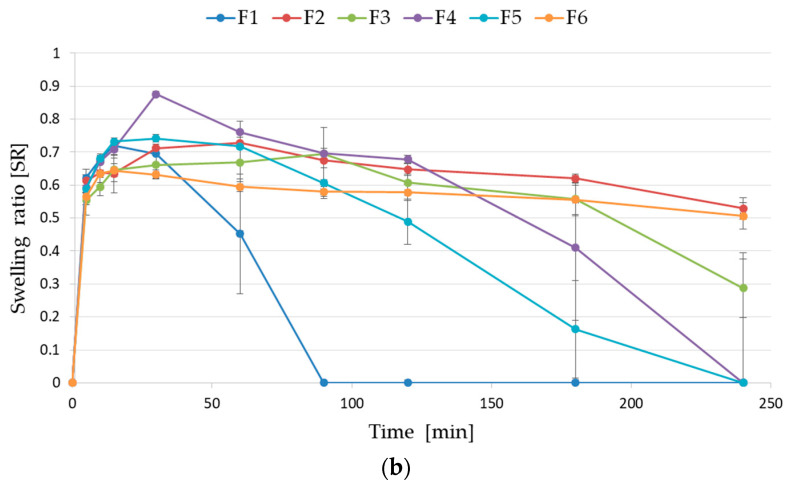
Swelling ratio of (**a**) formulation without active substance (P1–P6) and (**b**) formulation containing POS (F1–F6) for microcapsules in PBS at pH 6.8 (mean ± SD, *n* = 3).

Swelling analysis in the medium with alkaline pH imitating intestine conditions expressed that placebo formulations reached similar values as data obtained from tests conducted at an acidic pH. Formulation P1 achieved the highest value (0.795 ± 0.031), but formulation P5 achieved the lowest value (0.695 ± 0.029) after 240 min (Figure 7a). In contrast, POS-loaded formulations showed a significant decrease in the absorption capacity of the alkaline medium (Figure 7b). Microcapsules in formulation F1 composed only from ALG and cross-linked by 2% zinc acetate reached their maximum (0.719 ± 0.018) after 15 min of analysis and completely dissolved after 90 min. The highest SR value (0.876 ± 0.009) was achieved by the formulation F4, composed of the ALG/PEC combination F4, after 30 min (Figure 7b).

It was shown that as the cross-linking agent concentration increased, the microcapsules of all formulations were more stable and less prone to water absorption. This was also observed by Sharma et al., who indicated that the amount of absorbed water through calcium ALG nanoparticles decreased when the concentration of the calcium chloride applied as a cross-linking agent increased [30]. The higher content of the cross-linking factor resulted in more and smaller pores, which provided lower water absorption and, consequently, lower swelling [31]. Additionally, swelling properties were significantly dependent on the medium in which the research was conducted.

The addition of PEC to the formulation composition improved the swelling properties in the case of formulations cross-linked by 2% zinc acetate, which is related to the hydrophilic nature of PEC. Carbinatto et al. suggested that with the increase in the PEC content in the cross-linked PEC/high amylose starch matrices, the degree of their swelling increased [28]. However, it was demonstrated that the PEC content in the formulations obtained using the 3% and 5% cross-linking agents did not significantly improve the absorption capacity of the medium. This process might be due to the greater cross-linking in the microcapsule matrix [31].

The oral route is a common and convenient method of drug administration. However, it is characterized by many challenges regarding drug substance stability and ensuring an appropriate site of absorption. One direction to overcome these difficulties could be the implementation of mucoadhesive drug delivery systems. The utilization of the mucoadhesive process enables specific sites to be targeted in the gastrointestinal tract and to modulate active substance release. This process can ensure controlled plasma drug levels and the improvement of drug bioavailability [32].

**Figure 8:**
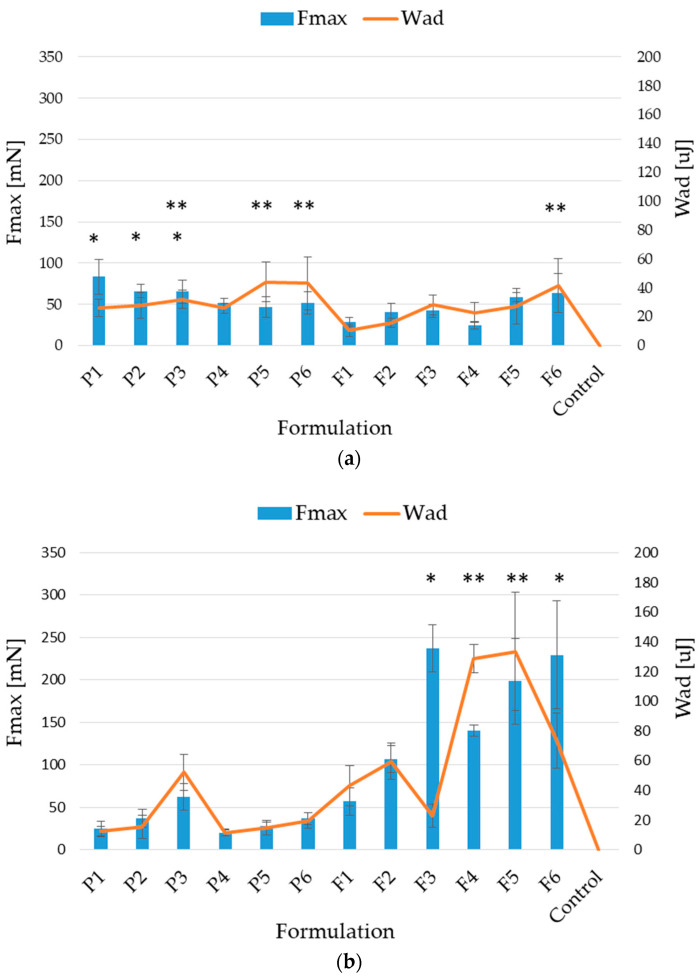
Mucoadhesiveness presented as force (F_max_) and work of adhesion (W_ad_) of formulation without active substance (P1–P6), containing POS (F1–F6) microcapsules and control (cellulose paper) using porcine stomach (**a**) and intestine mucosa (**b**) (mean ± SD, *n* = 6, significant statistical difference (*p* < 0.05) * of F_max_ and ** W_ad_ compared to correspondent value of F1).

Both polymers, ALG and PEC, demonstrate carboxyl groups in their chains, which might create hydrogen bonds with the hydroxyl groups of mucin glycoproteins. The consequential interaction of the polymers with mucous takes place [7,33]. Placebo and POS-containing formulations were analyzed for their mucoadhesive ability to two adhesive layer models: porcine stomach and intestinal mucosa. The analysis method was preceded by wetting the microcapsules with 0.1 M HCl (pH 1.2) or in PBS (pH 6.8) for 15 min before testing, followed by the experiment in the presence of a medium. The designed tests imitated in vivo conditions and eliminated adhesion due to water extraction from the moistened mucosa [17]. The mucoadhesive ability of the designed placebo and POS-loaded microcapsules are presented in Figure 8. It was indicated that all formulations interacted with applied layers, and mucoadhesiveness was influenced by the type of adhesive layer, the cross-linking agent concentration, and the presence of PEC.

The mucoadhesiveness analysis of prepared microcapsules using stomach mucosa showed that both the cross-linking process and the PEC presence slightly reduced F_max_, while in the case of W_ad_, an increase in this value was observed (Figure 8a). The highest value of F_max_ was noted in the formulation P1 (83.43 ± 20.71 mN) consisting of ALG only and cross-linked by 2% zinc acetate, but the highest values of W_ad_ were expressed for formulation P5 (44.15 ± 13.59 µJ). Microcapsules with drugs revealed decreased values of those parameters compared to formulations without an active substance. Interestingly, in the case of the POS-loaded formulation, an increase in the mucoadhesive capacity with the increasing cross-linking agent concentration and PEC presence was observed.

When porcine intestine mucosa was applied, the increase in F_max_ and W_ad_ was observed in the placebo and formulation containing POS (excluding formulations F3 and F6, where W_ad_ was significantly decreased) (Figure 8b). In POS-loaded formulations, a considerable improvement of F_max_ was recorded compared to data from the analysis mimicking the stomach environment. The highest values of F_max_ were expressed for formulations F3 (237.43 ± 27.83 mN) and F6 (229.56 ± 63.38 µJ).

Hagesaether et al. demonstrated that ZnCl_2_ cross-linking reduced the interaction of PEC with the mucosa. This probably occurs due to reducing the mobility of the polymer chains and, thus, limiting their ability to diffuse and interpenetrate mucin molecules [17]. Similar data were noted by Awasthi et al., who developed dual cross-linked PEC/ALG beads with repaglinide. They demonstrated that the beads’ mucoadhesiveness decreased with an increasing concentration of the cross-linking agent [34]. There are various mechanisms of mucoadhesion. Therefore, every formulation should be considered individually. In the featured study, the enhancement of the mucoadhesive characteristics of Zn^2+^ cross-linked ALG and ALG/PEC microcapsules was observed. This might be the result of the hydration in the first stage of testing the analyzed formulations, which may have caused the partial release of Zn^2+^ ions. The loss of the cross-linking agent led to the matrix loosening and the increased flexibility of the microcapsules. Then, positively charged cross-linking ions led to an improvement in the interactions with mucin anionic groups and the creation of adhesive bonds with the negatively charged tested layer [35,36]. The additional swelling step prior to analysis might also increase the adhesive properties of formulations containing POS.

The obtained data indicated that POS-loading formulations possessed a higher mucoadhesive ability in the intestine environment. It might provide more reproducible POS pharmacokinetics, enabling the maximization of absorption regardless of the food intake. Jelveghari et al. concluded that ALG/PEC microspheres cross-linked by CaCl_2_ due to their mucoadhesive properties to the intestinal mucosa provided significant protection for the tissues in the stomach and were a promising enteric formulation for piroxicam [37].

### 3.3. In Vitro POS Release

**Figure 9:**
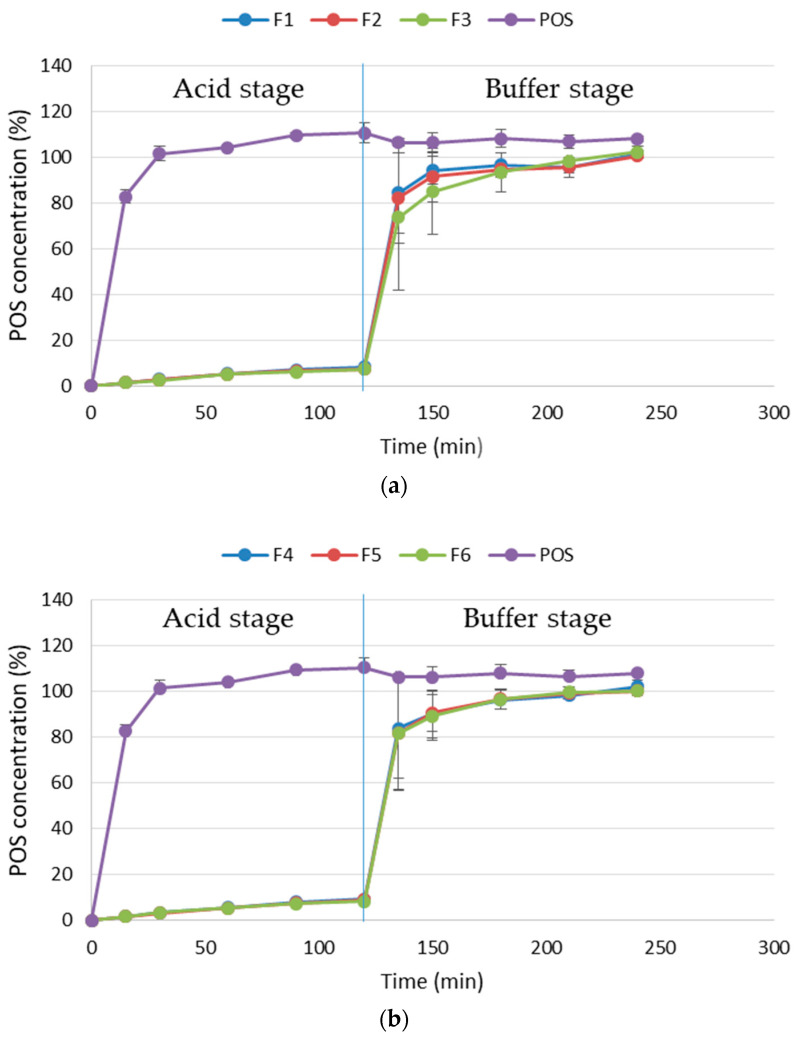
POS in vitro dissolution profile from formulations (**a**) F1–F3, (**b**) F4–F6, and control (POS—posaconazole powder) (mean ± SD, *n* = 3).

In vitro release profile analysis is a significant and important test, enabling a preliminary assessment of the availability of the drug from the developed drug formulation [38]. The in vitro POS release profile from ALG and ALG/PEC microcapsules cross-linked by zinc ions was conducted using the pH change pharmacopoeial protocol [18]. The acquired data are featured in Figure 9. It was demonstrated that in the case of both ALG and ALG/PEC POS-loaded formulations in an acidic medium imitating the stomach conditions (0.1 M HCl, pH 1.2), the POS concentration was below 10% over a period of 2 h and was within the scope of pharmacopoeial limits for enteric drug formulations [18]. The obtained results were comparable and independent of the cross-linking agent concentration (Figure 9).

In an alkaline environment, 80% of POS was released from all formulations after 15 min. The lower POS concentration in the medium was expressed for formulation F3, reaching 73.68 ± 31.65% after 15 min and then 102.18 ± 2.72% after 2 h of analysis (Figure 9a). This fact is related to the application of 5% zinc acetate. In the case of the formulation containing PEC, increasing the concentration of Zn^2+^ ions also resulted in the extension of the POS release profile, reaching the lowest concentration of the active substance in the case of formulation F6 (81.65 ± 24.93% after 15 min of testing in the alkaline medium), but the variations between the formulations were not considerable (Figure 9b). Differences in the POS release profiles were related to differences in the behaviour of ALG in the analyzed media. Below ALG pKa (3.6), upon contact with hydrochloric acid, ALG became swellable, but water-insoluble alginic acid had a tight three-dimensional network. This fact might affect the limited release of an active substance in an acidic environment. Additionally, under these conditions, Zn^2+^ ions form a strong three-dimensional network with ALG and retain the drug inside the microcapsules [39,40,41]. In the environment simulating intestinal conditions, the pKa value was higher than 3.6, and ALG negatively charged carboxylate ions were separate. This process can lead to the relaxation of the three-dimensional microcapsule network and the medium inflow. Then, phosphate ions displace Zn^2+^ ions from the cross-linked ALG and ALG/PEC matrix, causing its gradual dissolution and erosion [39,40,41]. As a result, the rapid release of POS in the alkaline environment simulating intestinal conditions was observed. pH-dependent drug release was also demonstrated by Auriemma et al., who developed gastro-resistant PEC and ALG beads cross-linked by zinc acetate containing betamethasone [40].

The active substance release from a developed formulation is a complex process. Understanding the mechanisms of this process is crucial when designing drug delivery systems, especially those releasing an active substance in a specific space and time [28]. Therefore, the data obtained from the evaluation of the release profile were subjected to mathematical analysis using zero and first-order kinetics equations and Highuchi, Hixson–Crowell, and Korsmeyer–Peppas models (Table 4).

The obtained values of the R^2^ correlation coefficient indicated that POS was released in accordance with first-order kinetics, depending on the concentration of all formulations. The analysis of the data according to the Highuchi and Hixson–Crowell laws indicated that the POS release process is related to both the diffusion of the active substance from the cross-linked polymer matrix and its erosion. However, the values of the diffusion coefficient of the Korsmeyer model reached 1.70 to 2.17 and expressed super case type II diffusion. Super case-II release is based on drug penetration by disentangling the swelling polymer chains and erosion of the matrix [42]. Similar results were obtained by Mankala et al. They observed that aceclofenac was released according to the case-II mechanism from mucoadhesive microcapsules prepared by the extrusion technique and composed of ALG combinations with cellulose derivatives [43].

### 3.4. Antifungal Activity

One of the frequently occurring fungal infections is candidiasis, caused by opportunistic fungi of the *Candida* genus. When immunity is weak, *Candida* species might cause not only dermatological but also systemic infections. The low-treatment efficacy of fungal diseases is associated with the limited number of available active substances and the occurrence of many drug-resistant fungal species [44]. Therefore, one of the therapeutic strategies is to search for new drug dosage forms that allow for effective and safe candidiasis treatment. Compared to traditional carriers, multi-compartment formulations might significantly improve the therapeutic effect through a shorter diffusion route and a larger surface area for the release of the drug substance.

**Figure 10:**
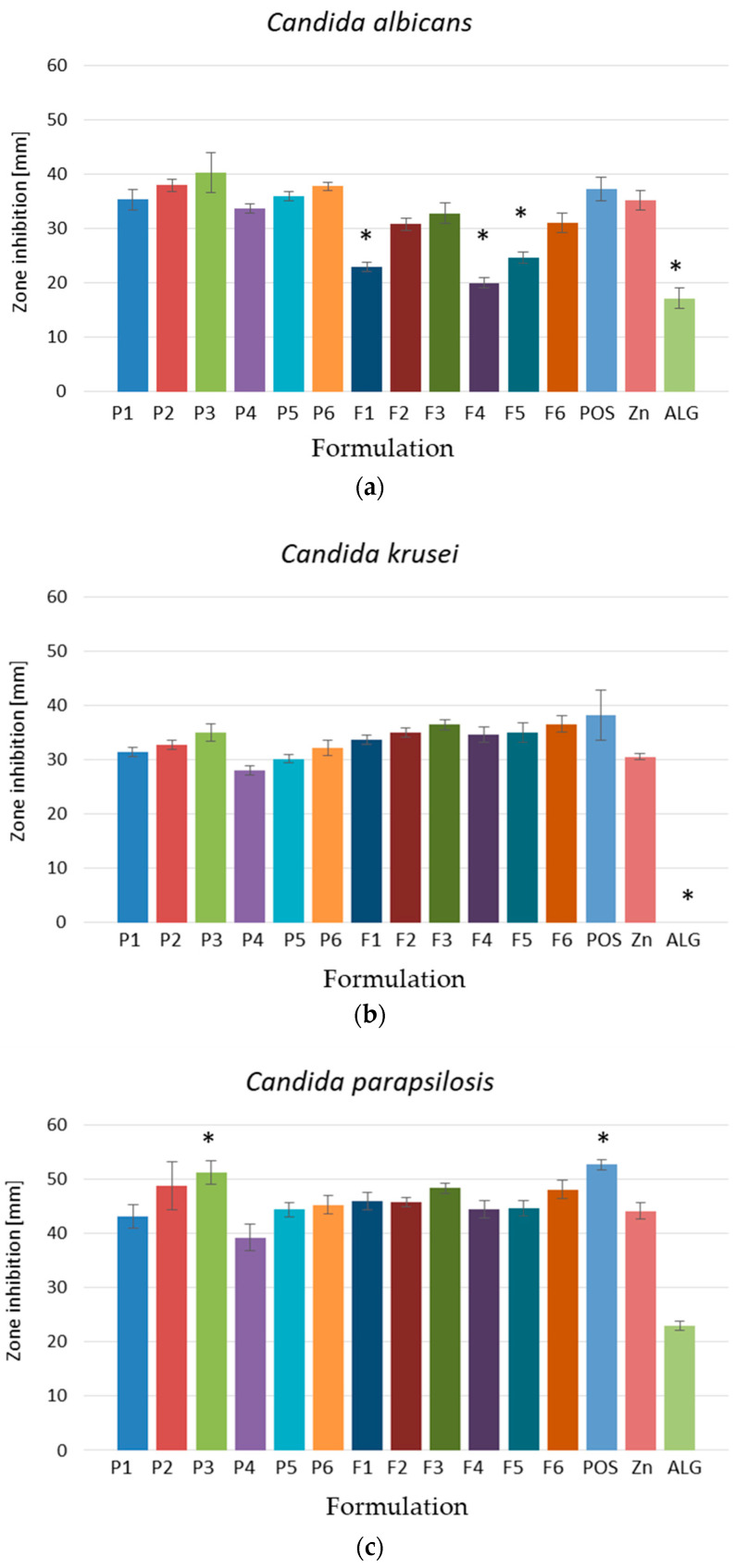
Antifungal effect of placebo of ALG and ALG/PEC microcapsule formulations (P1–P6), and formulations containing POS (F1–F6); POS (POS in DMSO), pure zinc acetate (Zn), and pure ALG for (**a**) *C. albicans*, (**b**) *C. krusei*, and (**c**) *C. parapsilosis* (mean ± SD, *n* = 6, * significant statistical difference (*p* < 0.05) versus to correspondent value of P1).

In order to determine the antifungal action of the obtained formulations, the disk diffusion procedure in accordance with CLSI guidelines was carried out [20]. The obtained data expressed that all formulations, both placebo and POS-loaded, possessed activity against the analyzed *Candida* strains (Figure 10). The most sensitive strain to the ALG and ALG/PEC microcapsules was *Candida parapsilosis*, where the highest inhibition zones (from 43.14 ± 2.19 mm in formulation P1 to 51.29 ± 2.14 mm in formulation P3) were recorded (Figure 10c). In the case of the POS-loaded formulation, the lowest sensitivity was noted for *Candida albicans* strains, while the highest was noted for *Candida parapsilosis* (Figure 10a,c). It was expressed that the strongest antifungal activity was characterized by the formulation composed of ALG only. The polymer negative charge facilitates interactions with fungal cells, leading to their rupture, the leakage of their interior contents, and, ultimately, microorganism death. Additionally, swelling ALG produces a polymer viscous layer surrounding the fungal or bacterial cells, which chelates ions [7]. This fact is confirmed by the data received during the antifungal analysis of pure ALG, which expressed significant antifungal action against *Candida albicans* and *Candida parapsilosis*, which reached 17.20 ± 1.92 mm and 23.00 ± 0.89 mm, respectively.

Although there are reports on the antimicrobial effects of PECs [16,45], surprisingly, the presence of PECs in ALG microcapsules cross-linked by Zn^2+^ ions resulted in a reduction in the antifungal action of all the analyzed *Candida* strains. However, it was recorded that the cross-linking agent possessed a significant impact on the increase in inhibition zones, which was dependent on the concentration of Zn^2+^ ions. Additionally, pure zinc acetate was characterized by an antifungal effect from 30.50 ± 0.58 mm against *Candida krusei* to 44.17 ± 1.47 mm against *Candida parapsilosis* (Figure 10b,c). The obtained results suggest that the utilization of Zn^2+^ ions as a cross-linking agent possesses a greater advantageous effect than the application of Ca^2+^ ions [16]. This was confirmed by Gong et al., who compared the antifungal effect of zinc, copper, and calcium alginate fibres. They indicated that zinc alginate fibres possess relevant antifungal activity against *Candida albicans* versus copper and calcium alginate fibres [46]. Similarly, Lakshmanan et al. discovered that the synthesized zinc/sodium alginate/polyethylene glycol/d-pinitol nanocomposites possessed strong antimicrobial properties against *Candida albicans* [47].

Zinc is involved in many important cellular reactions, is essential for proper development and cell division, and is a critical factor in the ageing process of the body and cell apoptosis. Additionally, it is characterized by antibacterial and antifungal effects [48]. Various mechanisms of zinc action have been proposed. An example includes the production of reactive oxygen species (ROS) such as the hydroxyl group, superoxide anion radicals, and hydrogen peroxide in microbial cells. Additionally, the direct contact of positively charged Zn^2+^ particles with differently charged cells could destabilize the microbial cell membranes, thereby limiting the growth of fungi in a dose-dependent manner [24,48]. The antimicrobial activity of zinc is utilized not only in dermatological preparations. There are randomized clinical trials expressing the beneficial clinical efficacy of Zn^2+^ ion supplementation in decreasing infections caused by *Candida* species in patients taking antibiotics [14].

### 3.5. Thermal Analysis

To assess the properties of designed formulations under different temperature conditions, the formulations were subjected to thermal analysis using thermogravimetry (TGA) and differential scanning calorimetry (DSC).

**Figure 11:**
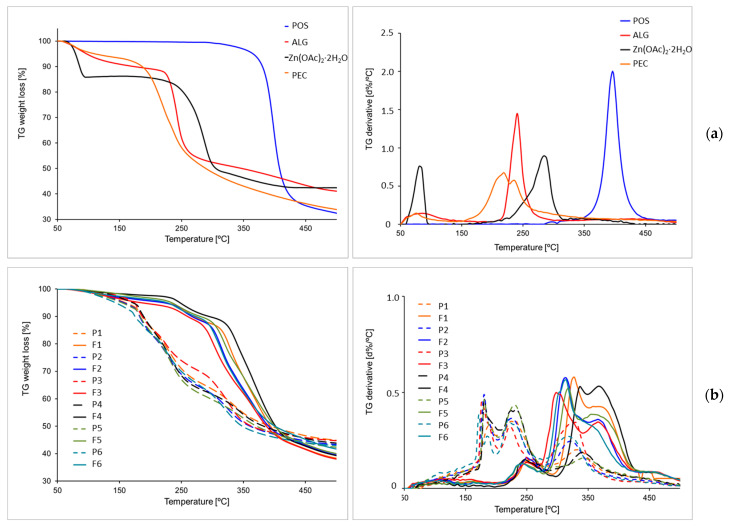
TGA and DTG curves of (**a**) pure components of microcapsules ALG, Zn(OAc)_2_·2H_2_O, PEC, and POS and (**b**) ALG and ALG/PEC microcapsules without active substances (P1–P6) and with POS (F1–F6).

Thermogravimetric analysis (TGA) is a technique that measures the mass of a sample over time as the temperature fluctuates. This analysis offers insights into physical processes like phase transitions, absorption, and desorption, along with chemical processes, including thermal degradation [49]. The TGA curves of all the samples, with the exception of POS, expressed three-step thermal degradation (Figure 11). Generally, the initial stage at 50–150 °C showed weight loss, which indicated the removal of moisture, the remains of solvents, or crystalline water, as in the case of zinc acetate (Figure 11).

TGA analysis showed that ALG was characterized by three decomposition temperature steps. The first step took place at 104 °C with 10.27% weight loss due to dehydration. The second stage, at 240 °C (47.37% weight loss), was the effect of the degradation of polymer glycosidic bonds in the process of decarboxylation and carbonization, and the third step (410 °C and 59.31% weight loss) indicated the transformation of ALG into Na_2_CO_3_ [50].

Pure PEC presented water loss at 90 °C in the first stage. The second step, which occurred between 230 °C and 440 °C, suggested polymer degradation. This process might be the result of the pyrolytic decomposition observed, consisting of primary and secondary decarboxylation involving the acid side group and the carbon ring [51,52]. On the other hand, the third stage represented polymer degradation with a total PEC mass loss of 58.34%.

Zinc acetate, similarly to ALG and PEC, presented three degradation steps involving the following temperatures: 80 °C, 280 °C, and 360 °C, demonstrating 57.52% total weight loss. However, POS demonstrated only one stage of degradation at 397 °C, reaching 28.08% of residues.

The analysis of the ALG and ALG/PEC placebo and POS-loaded microcapsules demonstrated that the thermal decomposition started at higher temperatures compared to the decomposition of pure excipients. The TGA curves of all formulations showed a small mass loss of up to 150 °C, which was related to water evaporation. It was observed that all formulations (except F5 and F6) were characterized by a three-stage decomposition. The first stage took place in a range from 50 °C to 210 °C. The weight loss in this range varied from 3% for formulation F4 to 16% for F1. The second step of degradation was observed in the range from 160 °C to 300 °C, with a weight loss of 6% (formulations F3 and F6) to 33% (formulation F4). The third stage was noted between 260 °C and 500 °C. All microcapsule formulations expressed a similar total weight loss ranging from 55% (formulation F1) to 63% (formulation F3). POS-loaded microcapsules expressed the maximum DTG peak (the first derivative of d%/°C) at higher temperatures than the placebo, which was probably related to the decomposition of the POS content at 397 °C. Based on the received data, it can be deduced that both ALG and ALG/PEC microcapsules cross-linked by zinc acetate are characterized by thermal stability.

**Figure 12:**
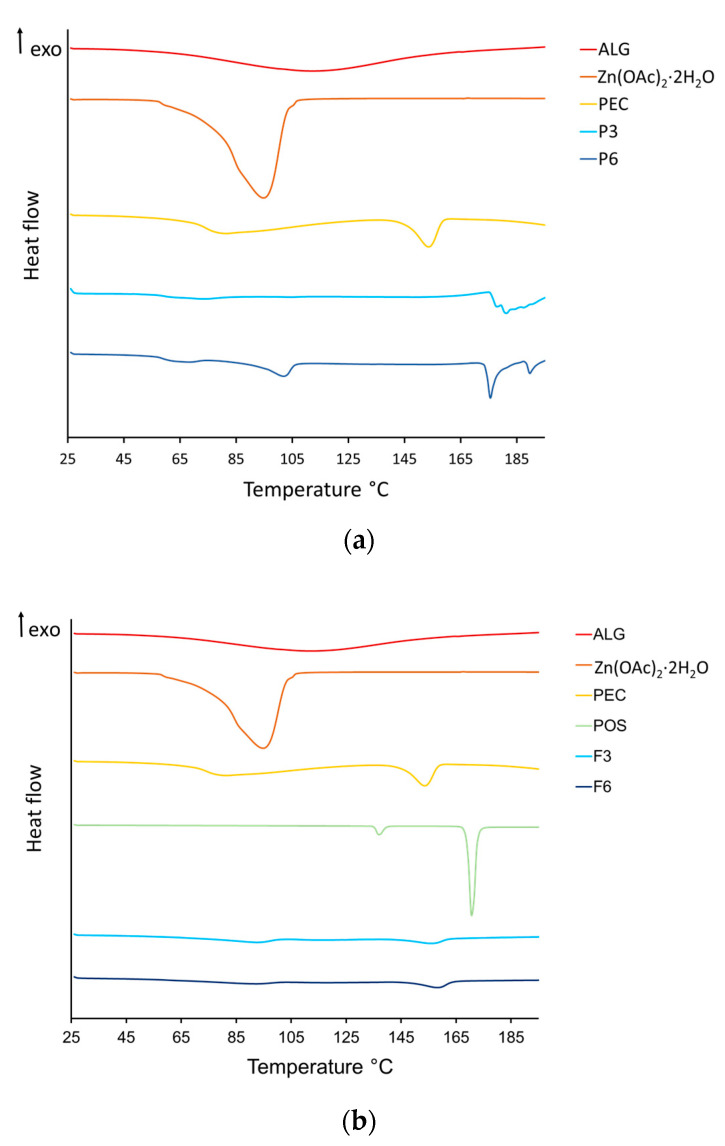
Representative thermograms of unprocessed ALG, Zn(OAc)_2_·2H_2_O, PEC, and POS: (**a**) microcapsule formulations P3 and P6 without drugs and (**b**) formulations F3 and F6 containing POS.

The next step in thermal analysis was the DSC study of the prepared placebo, POS-loaded formulations, and their individual pure ingredients. Figure 12 demonstrates representative thermograms. The thermogram of pure ALG expressed a broad endothermic peak in the range of 48.33–165.03 °C. This peak is related to the removal of water. From 48.33 °C to 60 °C, elimination of free-release ALG water was noted; from 120 °C, water interacting with the hydroxyl groups of ALG was eliminated; and in the range of 160–165.03 °C, water bound to the carboxylic acid groups of the polymer was eliminated (Figure 12a) [53]. This fact was confirmed by the ALG mass loss in this temperature range in TG analysis (Figure 11). The PEC thermogram presents two endothermic peaks. The first inconsiderable peak is at 81.50 °C, which can be related to the loss of bound water. The other pronounced peak was detected at 154.50 °C. This is probably associated with the change in the conformation of the polymer galacturonan ring chair [54]. The endothermic peak in the range of 58 to 110 °C zinc acetate was related to the removal of crystalline water, which was also confirmed by TG analysis (Figure 11 and Figure 12). The calorimetric analysis of POS presented two endothermic peaks—a slight peak at 137.50 °C and a sharp peak at 170.50 °C—which correspond to the literature melting point range (170–172 °C) [47]. The presence of peaks improved the purity of the active substance. The DSC curves of formulations without drugs presented a slight shift and reduction in endothermic peaks of ALG and PEC. In cross-linked formulations, a small peak originating from zinc acetate was observed (Figure 12b). In DSC thermograms of microcapsules containing POS, no ALG, POS, or Zn^2+^ peaks were noted. This fact indicated the formation of an amorphous POS, which might be related to the presence of the solubilizer (polysorbate 80) in the formulation [55]. Amorphous substances, in comparison to crystalline forms, possess greater stability, solubility, and biological availability [56].

### 3.6. Attenuated Total Reflectance–Fourier Transform Infrared Spectroscopy (ATR–FTIR)

Attenuated Total Reflectance–Fourier Transform Infrared Spectroscopy (ATR–FTIR) is an analytical technique utilized to identify functional groups in a molecule, assess the structure of molecules, and determine the composition of chemical compounds. Additionally, ATR–FTIR, by determining the signals of functional groups and assessing the changes, frequencies, shifts, and intensities in their bands, enables the detection of drug–polymer interactions [57].

**Figure 13:**
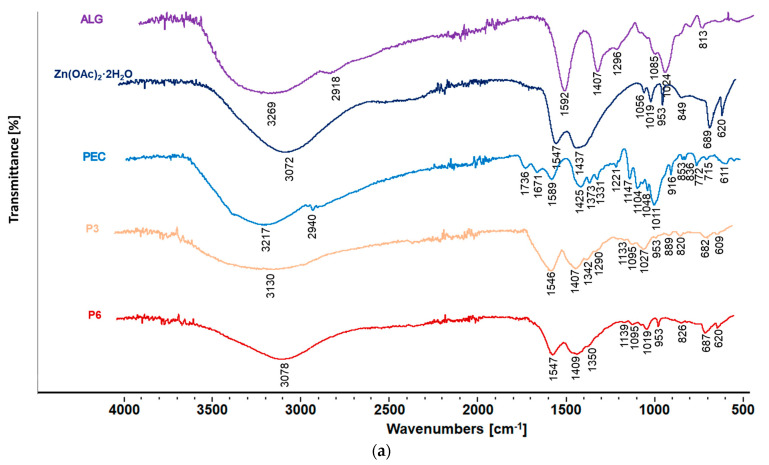

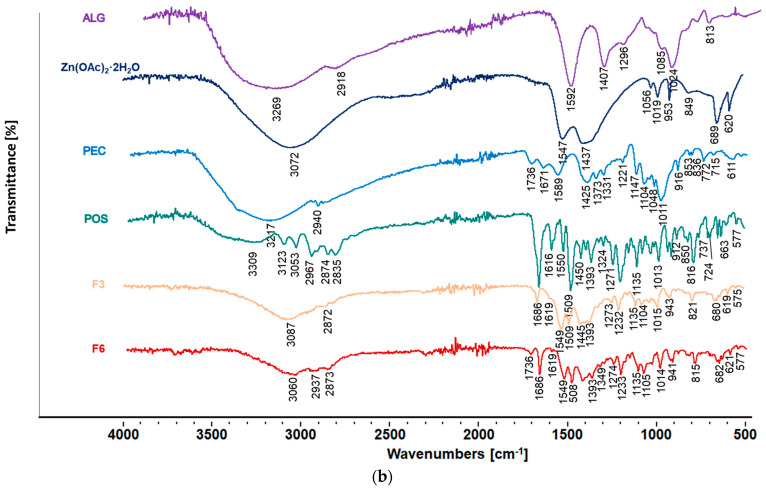
Representative FTIR-AR spectra of unprocessed ALG, Zn(OAc)_2_·2H_2_O, PEC, POS: (**a**) microcapsule formulations P3 and P6 without drugs and (**b**) formulations F3 and F6 containing POS.

The ATR-FTIR analysis of the designed formulations is presented in Figure 13. It is shown that the presence of ALG bands (1294, 1407, 1296, 1085, and 1024 cm^−1^) is related to the vibrations of carboxylic carbonyl groups. Additionally, the broadened band at 3500–2800 cm^−1^ in the spectra of sodium alginate was noted, which indicated the stretching vibration of the OH bond [13]. In the ATR-FTIR spectra of formulation P4-P6, additional bands originating from PEC (1736 cm^−1^ and 1147 cm^−1^) were demonstrated (Figure 13a). The analysis of cross-linked formulations of both placebo and POS-loaded formulations indicated the presence of zinc acetate bands in the wavelength range of 1547, 953, and 689 cm^−1^. Moreover, when the percentage of Zn^2+^ ions in the formulations increased, an increase in the intensity of the band originating from the vibrations of the Zn-O bond at approx. 680 cm^−1^, and an increase in the band intensity in the range of 3050–3090 cm^−1^ was visible. During the cross-linking process, the carbonyl-stretching vibrations shifted towards a lower wave number. This was the result of the coordination bond between ALG and Zn^2+^ ions [13]. The ATR-FTIR spectra of POS-loaded formulations (F1–F6) were completely dominated by the bands originating from the bond vibrations of the active substances. Additionally, an increase in the band intensity in the range of 3050–3090 cm^−1^ was visible with the increase in the amount of zinc acetate applied in the cross-linking process (Figure 13b). The presence of POS bands in the formulation indicated the successful encapsulation of POS in ALG and ALG/PEC microcapsules without chemical changes. This fact also expressed the absence of drug–polymer interactions during the encapsulation process.

## 4. Conclusions

The co-extrusion method enabled ALG and ALG/PEC microcapsules cross-linked with zinc acetate containing an antifungal substance—POS—to be received. All prepared formulations were characterized by a regular shape, uniformity of size, relatively high encapsulation efficiency, production yield, and the content of active substances. It was shown that the cross-linking process with Zn^2+^ ions allows drug dosage formulations to be received, demonstrating their stability in acidic environments and ability to release the total POS content in conditions simulating the intestinal environment within 2 h. Additionally, the application of Zn^2+^ ions as a cross-linking agent significantly influenced the antifungal activity against the analyzed *Candida* strains. However, the presence of PEC in the formulation reduced the firmness of the microcapsules, caused increased swelling in an environment mimicking intestinal conditions, and significantly improved the mucoadhesive properties of POS-loaded formulations to the intestinal mucosa.

## Figures and Tables

**Table 1:** Contents of microcapsules ALG and ALG/PEC placebo and POS-loaded formulations.

Formulation	ALG [%]	PEC [%]	POS [%]	Zn^2+^ [%]	Polysorbate 80 [%]
P1	2	–	–	2	1
P2	2	–	–	3	1
P3	2	–	–	5	1
P4	1	1	–	2	1
P5	1	1	–	3	1
P6	1	1	–	5	1
F1	2	–	5	2	1
F2	2	–	5	3	1
F3	2	–	5	5	1
F4	1	1	5	2	1
F5	1	1	5	3	1
F6	1	1	5	5	1

**Table 2:** Features of the ALG and ALG/PEC mixtures both without and containing POS (*n* = 3).

	pH	Viscosity (mPa∙s)
2% ALG	8.02 ± 0.02	2413.33 ± 40.41
2% ALG/5% POS	7.60 ± 0.06	233.33 ± 20.82 *
1% ALG/1% PEC	4.92 ± 0.09 *	343.33 ± 5.77 *
1% ALG/1% PEC/5% POS	4.73 ± 0.05 *	287.0 ± 6.08 *

* Significant differences (*p* < 0.05).

**Table 3:** Quality of empty microcapsules (P1–P6) and formulation with POS (F1–F6).

Formulation	Diameter (mm)		Production Yield (%)	Drug Loading (%)	Encapsulation Efficiency (%)
	Wet	Dry			
P1	2.85 ± 0.26	1.24 ± 0.03	90.04 ± 4.66 *	–	–
P2	3.12 ± 0.21	1.15 ± 0.05 *	98.13 ±1.80 *	–	–
P3	3.35 ± 0.16	1.00 ± 0.05 **	75.64 ± 3.57 *	–	–
P4	3.16 ± 0.10	1.15 ± 0.02 *	68.33 ± 8.13 *	–	–
P5	3.09 ± 0.08	1.07 ± 0.03 **	58.47 ± 1.27 *	–	–
P6	3.09 ± 0.18	0.94 ± 0.03 **	57.31 ± 0.86 *	–	–
F1	3.09 ± 0.08	1.50 ± 0.02	39.54 ± 1.12	73.81 ± 3.68	103.35 ± 5.15
F2	2.99 ± 0.07	1.48 ± 0.04	40.12 ± 0.60	74.24 ± 4.19	103.95 ± 5.87
F3	3.02 ± 0.13	1.48 ± 0.02	38.38 ± 1.10	69.04 ± 6.48 *	96.67 ± 9.07 *
F4	3.03 ± 0.08	1.24 ± 0.04	39.13 ± 0.48	82.07 ± 4.98 *	114.92 ± 6.97 *
F5	2.97 ± 0.08	1.21 ± 0.04	34.12 ± 0.48 *	79.26 ± 2.21	110.98 ± 3.10
F6	2.88 ± 0.05 *	1.18 ± 0.06 **	33.92 ± 0.40 *	71.17 ± 1.48	99.65 ± 2.08

Significant statistical differences at * (*p* < 0.05) and ** (*p* < 0.001) compared to the correspondent value of F1.

**Table 4:** Models of POS release from ALG and ALG/PEC microcapsules.

Formulation	Zero-Order Kinetics		First-Order Kinetics		Highuchi Model		Hixson–Crowell Model		Korsmeyer–Peppas Model		
	R^2^	K	R^2^	K	R^2^	K	R^2^	K	R^2^	K	*n*
F1	0.78	0.56	0.84	0.02	0.73	10.61	0.71	0.02	0.81	1.20	1.70
F2	0.78	0.55	0.84	0.02	0.74	10.50	0.71	0.02	0.82	1.29	1.75
F3	0.78	0.55	0.85	0.02	0.76	10.47	0.72	0.02	0.82	1.29	2.17
F4	0.79	0.56	0.85	0.02	0.75	10.60	0.72	0.02	0.82	1.30	1.91
F5	0.79	0.56	0.85	0.02	0.75	10.58	0.72	0.02	0.82	1.30	1.96
F6	0.79	0.56	0.84	0.02	0.74	10.58	0.98	0.03	0.82	1.30	1.99

## Data Availability

The data are contained within the article; raw data are available upon request.
